# Conservation energetics of beluga whales: using resting and swimming metabolism to understand threats to an endangered population

**DOI:** 10.1242/jeb.246899

**Published:** 2024-03-14

**Authors:** Jason S. John, Dennis R. Christen, Katherine L. Flammer, Traci L. Kendall, Emily C. Nazario, Beau P. Richter, Verena Gill, Terrie M. Williams

**Affiliations:** ^1^ University of California, Santa Cruz, Department of Ecology and Evolutionary Biology, 130 McAllister Way, Santa Cruz, CA 95060, USA; ^2^ Georgia Aquarium, 225 Baker Street Northwest, Atlanta, GA 30313, USA; ^3^ NOAA Fisheries, 222 W. 7th Ave, Anchorage, AK 99501, USA

**Keywords:** Field metabolic rate, Cost of transport, Accelerometry, Marine mammals, Cetaceans, Dolphins, Metabolic rate, Diving

## Abstract

The balance between energetic costs and acquisition in free-ranging species is essential for survival, and provides important insights regarding the physiological impact of anthropogenic disturbances on wild animals. For marine mammals such as beluga whales (*Delphinapterus leucas*), the first step in modeling this bioenergetic balance requires an examination of resting and active metabolic demands. Here, we used open-flow respirometry to measure oxygen consumption during surface rest and submerged swimming by trained beluga whales, and compared these measurements with those of a commonly studied odontocete, the Atlantic bottlenose dolphin (*Tursiops truncatus*). Both resting metabolic rate (3012±126.0 kJ h^−1^) and total cost of transport (1.4±0.1 J kg^−1^ m^−1^) of beluga whales were consistent with predicted values for moderately sized marine mammals in temperate to cold-water environments, including dolphins measured in the present study. By coupling the rate of oxygen consumption during submerged swimming with locomotor metrics from animal-borne accelerometer tags, we developed predictive relationships for assessing energetic costs from swim speed, stroke rate and partial dynamic acceleration. Combining these energetic data with calculated aerobic dive limits for beluga whales (8.8 min), we found that high-speed responses to disturbance markedly reduce the whale's capacity for prolonged submergence, pushing the cetaceans to costly anaerobic performances that require prolonged recovery periods. Together, these species-specific energetic measurements for beluga whales provide two important metrics, gait-related locomotor costs and aerobic capacity limits, for identifying relative levels of physiological vulnerability to anthropogenic disturbances that have become increasingly pervasive in their Arctic habitats.

## Teaser

**Highlighted Article:** Elevated energetic costs for rest and continuous locomotor activity by beluga whales limit underwater escape from anthropogenic threats.

## INTRODUCTION

Of the 77 species of small to moderately sized toothed whales, 98% show unknown or declining population trends (www.iucnredlist.org) that have been attributed to increasing global anthropogenic threats in the oceans (e.g. Duarte et al., 2021). The problem has been exacerbated by the difficulty of studying free-ranging cetaceans in order to both understand and mitigate the threats. The Cook Inlet beluga whale population is of particular concern owing to its small population numbers and proximity to anthropogenic activities (NMFS, 2016). Designated as a distinct population segment because of its geographic isolation from other beluga whale populations, the Cook Inlet beluga whale was listed as endangered under the Endangered Species Act (ESA) in 2008 after significant depletion resulting from unregulated subsistence hunting. Despite cessation of hunting and protections under the ESA, this population has failed to recover and is currently estimated at 279 individuals (Shelden and Wade, 2019).

**List of symbols and abbreviations**

ADL: aerobic dive limit
BMR: basal metabolic rate
cADL: calculated aerobic dive limit
COT_TOT_: total cost of transport
ESA: Endangered Species Act
FMR: field metabolic rate
*f*_S_: swimming stroke rate (frequency)
LML: Long Marine Laboratory
*M*: body mass
NOAA: National Oceanic and Atmospheric Agency
NMFS: National Marine Fisheries Service
ODBA: overall dynamic body acceleration
PDBA: partial dynamic body acceleration
RMR: resting metabolic rate
*S*_ss_: number of strokes for submerged swimming
*U*_swim_: swim speed
*V̇_O2_*: rate of oxygen consumption
*V̇_O2ss_*: rate of oxygen consumption during submerged swimming
*V̇_O2_*_surfswim_: rate of oxygen consumption during surface swimming

With such a small, vulnerable population, an important tool in the conservation management of beluga whales, as with other wild animals, is bioenergetic modeling (Cooke et al., 2014; Wikelski and Cooke, 2006), which can help to identify the species' physiological capacity to respond to anthropogenic disturbances (Costa, 2012; Kendall et al., 2013; Lotze et al., 2017; McHuron et al., 2017; Pirotta et al., 2019; Williams et al., 2017a), and consequent demands on environmental resources (Estes, 1996; Williams et al., 2011a, 2004a; Roman et al., 2014). The effectiveness of these models for marine vertebrates depends on detailed knowledge of key energetic variables. Most importantly, these include accurate resting metabolic rates (RMRs) to set the energetic foundation, and active metabolic costs for locomotion (Costa, 2012; Pirotta et al., 2018a,b) to identify demands related to transiting, foraging, as well as avoidance maneuvers and escape from disturbance. The former provides a baseline for comparing the unique energy requirements of different species, and for evaluating energetic balance from individuals to populations (Costa, 2012). The latter, when combined with resting energetic costs, can then be used to estimate field metabolic rates (FMRs) (Noren, 2011; Williams et al., 2004a), and likewise, the prey biomass needed to sustain the energetic needs of populations (Benoit-Bird, 2004; Rosen and Trites, 2013; Bejarano et al., 2017). Both underlie critical aspects of species management plans (Noren, 2011; Rechsteiner et al., 2013; Hays et al., 2019).

Such energetic metrics are currently lacking for many species of marine mammal. This is especially evident for medium-sized cetaceans such as beluga whales compared with other marine mammal groups (i.e. pinnipeds, sea otters and polar bears). To date, most metabolic and energetic research on cetaceans has focused on smaller species such as harbor porpoises (approximately 50 kg, *Phocoena phocoena*; Worthy et al., 1987; McDonald et al., 2017; Rojano-Doñate et al., 2018) and bottlenose dolphins (approximately 200 kg, *Tursiops truncatus*; Peddemors, 1990; Yazdi et al., 1999; Williams et al., 1999, 2017b; Miedler et al., 2015). Among larger odontocetes, metabolic rates have been directly measured for killer whales (3500 kg, *Orcinus orca*; Kriete, 1995; Worthy et al., 2014; Williams et al., 2017b), and beluga whales with conflicting results for resting metabolism as described below.

The RMRs of trained beluga whales (*Delphinapterus leucas*) have been reported for one adult male (Rosen and Trites, 2013), one pregnant adult female and juveniles of both sexes (Kasting et al., 1989). RMR levels from these studies differed from each other beyond what can be explained by sex or reproductive status. In another study, the respiratory rate of trained beluga whales was used as a proxy for metabolic demand (George and Noonan, 2014). Currently, the accuracy of this method is unknown, particularly when variation in breathing volume and duration is taken into account (Roos et al., 2016). Another respiratory-based study with beluga whales found a paradoxical decrease in respiration rates with increasing swim speed, demonstrating the need for higher resolution measurements over extended durations (Shaffer et al., 1997).

In view of the inconclusive metabolic data for beluga whales and the need for consistent, accurate data for management models, the goal of the present study was to measure the energetics of resting and active beluga whales under controlled conditions. These data were compared with those of a smaller, routinely studied odontocete, the Atlantic bottlenose dolphin. The energetic profiles of these odontocetes, in turn, were compared with those previously reported for other marine mammals, including sea otters, otariids and phocid seals, using different forms of swimming locomotion. We measured the metabolic rates of the cetaceans during sedentary stationing on the water surface (RMR), as well as during submerged swimming, to determine average swimming cost, cost per stroke and total cost of transport (COT_TOT_). Oxygen consumption data from the beluga whales were then matched with locomotor metrics collected with animal-borne accelerometer tags and visual observations. These were compiled to determine stroke-by-stroke locomotor costs and to develop predictive metrics for assessing energy expenditure in wild beluga whales. In this way, the study was designed to measure and test the physiological data required to create predictive energetic models to evaluate the impacts of anthropogenic disturbance on wild beluga whales.

## MATERIALS AND METHODS

### Animals and experimental design

We measured resting and active metabolic rates on one young adult male and two young adult female beluga whales [*Delphinapterus leucas* (Pallas 1776)] housed at the Georgia Aquarium (Atlanta, GA, USA), and two young adult male Atlantic bottlenose dolphins [*Tursiops truncatus* (Montagu 1821)] at the Long Marine Laboratory (LML, Santa Cruz, CA, USA) (Table 1). Age classifications were based on growth curves from wild animals (beluga: Vos et al., 2019; dolphin: Bejarano et al., 2017; Cheney et al., 2018), with the cetaceans in the present study within 90–100% of the adult plateau region for growth in these species. In view of this and the overlap in the range of body masses with adults, we assumed that potential growth-related effects on metabolic rate were minimal. Trials were conducted in temperature-controlled, saltwater pools with a maximum depth of 7.3 m for beluga whales and 9.1 m for dolphins, with average water temperatures of 15°C for beluga whales and 20°C for dolphins. Owing to the comparatively shallow depths and constant stroking behaviors of the animals, we have classified these trials as ‘submerged swimming’ rather than ‘diving’ per se, which may entail prolonged gliding periods (Williams et al., 2022) and depth-related cardiovascular changes (Williams et al., 2015) in free-ranging cetaceans.

**Table 1.**
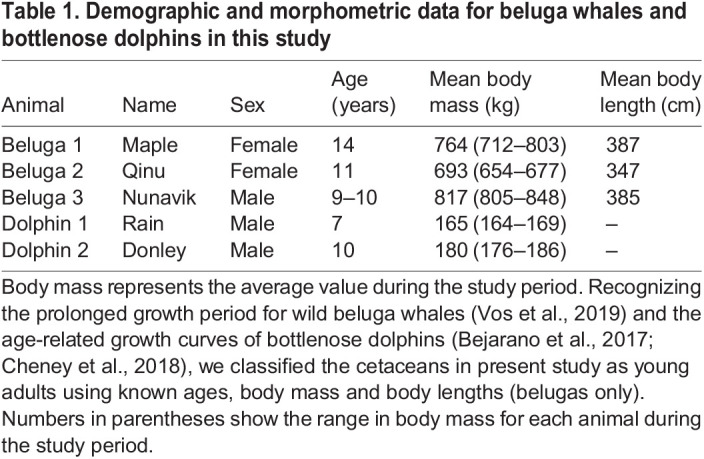
Demographic and morphometric data for beluga whales and bottlenose dolphins in this study

Both species were fed a mixed fish diet. Training for both active and resting behaviors began more than 6 months before data collection using positive reinforcement and operant conditioning techniques. The metabolic measurement period was from November 2018 to November 2019 for beluga whales and from March 2019 to March 2020 for the dolphins. Resting measurements were taken in individual trials throughout these periods, with the average individual RMR used as a target baseline for each animal during recovery from submerged swimming. Active measurements were taken during fasted conditions for dolphins (>8 h since the last feeding), and during both fasting and fed conditions for beluga whales to determine the effect of both states on swimming metabolic costs. All procedures were approved by the Georgia Aquarium Institutional Research Committee and the University of California, Santa Cruz Institutional Care and Use Committee following the National Institutes of Health guidelines. The research was conducted under Marine Mammal Protection Act permits issued through the NOAA Fisheries Office of Protected Resources.

**Fig. 1.**
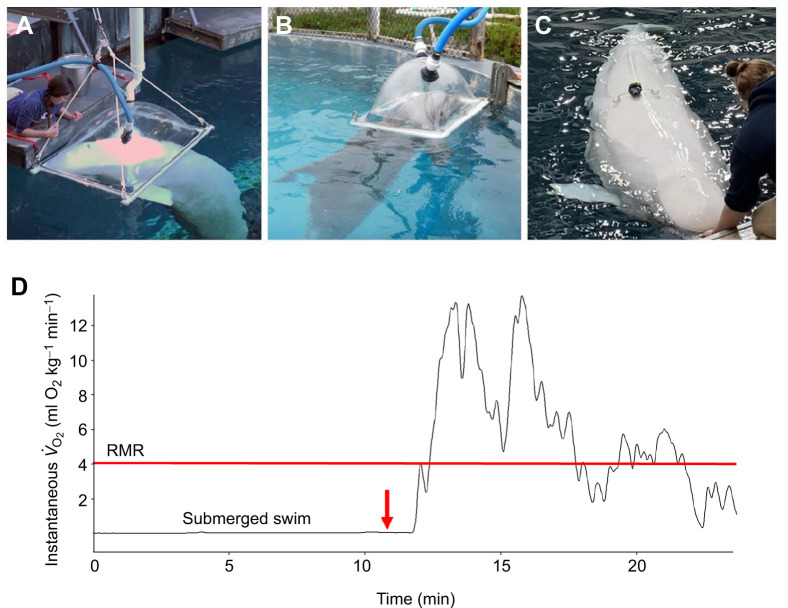
**Metabolic chambers for measuring oxygen consumption.** (A) Beluga whales at Georgia Aquarium and (B) bottlenose dolphins at University of California, Santa Cruz. Both resting and post-submerged swim recovery behaviors were trained for at least six months prior to data collection to minimize extraneous behaviors during experimental trials. (C) The placement of an accelerometer tag along the dorsal ridge of a beluga whale. (D) A representative trace for instantaneous oxygen consumption in relation to time is shown for a sedentary beluga whale (beluga 1) following a fast submerged swim. Arrival in the respiratory dome (red arrow) initiates the period of post-swim recovery to eventual resting metabolic rate (RMR; red line). Variation in instantaneous *V̇*_O_2__ is due to respiratory patterns of the animal.

We determined energy expenditure from measurements of oxygen consumption using open-flow respirometry. Measurements were taken using a plexiglass metabolic dome (beluga whale: 127×81×36 cm; dolphin: 85×58×36 cm; length×width×height) mounted on the water surface (Fig. 1A,B). Stroke mechanics and acceleration were measured simultaneously during swims with three-axis accelerometers (Fig. 1C; CATS-Diary, Customized Animal Tracking Solutions, Oberstdorf, Germany) placed on belugas 2 and 3. For beluga 1, strokes were counted manually by observers stationed at underwater windows using both video (30 frames s^−1^) and visual recordings. Metabolic rate during surface resting was measured in all animals, and submerged swimming was measured in all beluga whales and dolphin 1.

### Resting metabolic rate

RMR was determined during steady-state sedentary behavior while the cetaceans voluntarily floated on the water surface. The animals stationed dorsal side up under the metabolic dome for 10–15 min with minimal movement (Fig. 1). All resting measurements were performed under fasted conditions. For these trials, the animals were first moved into position beside the metabolic dome for 1–3 min before data collection to prevent movement costs from influencing resting measurements after the animals floated into the metabolic dome.

### Energetic cost of submerged swimming

The whales and dolphins were trained to remain submerged >1–2 m below the water surface depending on species to prevent surface drag (Fish, 1994) and swam a measured loop circuit to maximum depth of the pools (beluga whale: 34 m lap length; dolphin: 59 m lap length) with continuous stroking until recalled to the metabolic dome by the trainer. Fasted swims were performed >8 h after last feeding. Fed swims for beluga whales were performed <2 h after ingesting approximately 4–5 kg of fish. Swim speeds represented the preferred voluntary speed for each animal, with exercise duration ranging from 1.5 to 3.0 min for dolphins and 2.0 to 4.0 min for beluga whales on each trial to approximate typical diving behaviors (Goetz et al., 2012a,b). Following the swim, the animals were recalled and signaled to surface inside the metabolic dome for measurement of recovery oxygen consumption.

### Data collection and analysis

#### Oxygen consumption and energetics assessments

Oxygen consumption was measured via open-flow respirometry using protocols from Williams et al. (2004b, 2017b). The animals were trained to only breathe under the plexiglass metabolic dome mounted on a PVC frame that floated on the water surface (Fig. 1). Air was pulled through the dome with a calibrated vacuum pump (FlowKit Mass Flow Generator, Sable Systems International, North Las Vegas, NV, USA) at a rate of 500 l min^−1^ for beluga whales and 300 l min^−1^ for dolphins for a response time of 1 min by the respirometry system. Environmental air temperature ranged from 20°C to 27°C during the trials. Air flowrate was regulated and subsampled for oxygen content using a mass flow controller and oxygen analyzer (FoxBox Respirometry System, Sable Systems International). Prior to oxygen analysis, subsamples were passed through a series of six tubes filled with desiccant (﻿Drierite, W. A. Hammond Drierite, Xenia, OH, USA) and CO_2_ absorbent (﻿Sodasorb, W. R. Grace & Co, Chicago, IL, USA). Subsampled oxygen content was continuously monitored and recorded at 1 Hz on a laptop computer using Expedata Analysis software (Sable Systems International). These values were corrected for standard temperature and pressure and converted to *V̇*_O_2__ assuming a respiratory quotient of 0.77 (Davis et al., 1985) and using equations from Withers (1977) and Fedak et al. (1981). The system was calibrated before each data collection period using dry ambient air (20.95% O_2_) and weekly with N_2_ gas according to the protocols of Fedak et al. (1981) and Davis et al. (1985). All values are reported in J kg^−1^ min^−1^ using a conversion factor of 20.1 J ml^−1^ O_2_ for comparison with previous studies (Williams et al., 2017b).

For RMR measurements, the lowest mean oxygen consumption for a minimum of 5 min was recorded for each trial. This minimum allowed the animals and respirometry system to reach steady state (1–3 min) and accounted for variations owing to apneic periods that could reach 20 s during the measurements. Thus, total measurement time after stabilization ranged from 5 to 12 min during each trial. Submerged swimming metabolic rates (*V̇*_O_2_ss_, J kg^−1^ min^−1^) were measured by calculating the total post-submergence oxygen consumed during recovery that was in excess of RMR following the procedures of Williams et al. (2004b, 2017b). This required first determining the baseline post-absorptive oxygen consumption rates for each whale or dolphin while resting on the water surface in separate trials. Return to this baseline value was used to define the duration of each post-submergence recovery period. Total target post-swim metabolic measurement time was 12 min to ensure return to baseline levels. Oxygen consumption was monitored continuously during the recovery period with the submerged metabolism calculated from the difference between recovery oxygen consumed and the baseline resting rates (see Fig. 1D).

#### Acceleration

Acceleration was measured in beluga whales using submersible tri-axial accelerometers recording in m s^−2^ at 20 Hz and converted to ***g*** (1 ***g***=9.81 m s^−2^). The three axes measured were defined as longitudinal or caudal–rostral acceleration (*x*-axis), dorso-ventral acceleration (*y*-axis) and lateral acceleration (*z*-axis). The accelerometer was attached along the dorsal center line immediately forward of the dorsal ridge using a suction cup mount (Fig. 1C), representing a common site for tag deployment on free-ranging cetaceans. Anatomical location of the tag was identical for all beluga whales in this study. The frontal area of the CATS-Diary accelerometer and mount was approximately 60 cm^2^ (<2% of the frontal surface area of the whales) and streamlined to avoid excess hydrodynamic drag during swimming (Kyte et al., 2019). Desensitization training started 6 months before data collection to prevent the tag attachment from influencing swimming mechanics or behavior.

#### Stroke mechanics and cost of transport

Because individual swimming trials were short compared with those of free-ranging cetaceans, swimming strokes could be manually counted from deflections in the *x*-axis data from the accelerometer when graphed. Total number of strokes per submerged swim (*S*_ss_) was determined using *x*-axis acceleration (longitudinal axis) and counting a full stroke cycle as one individual stroke. *S*_ss_ was then divided by the total submergence time in minutes to calculate stroke frequency (*f_S_*, strokes min^−1^) for each trial. Dividing the total energy expended during the swim (J kg^−1^) by *S*_ss_ enabled us to define the cost per stroke (J kg^−1^ stroke^−1^). Lastly, the total cost of transport (COT_TOT_, J kg^−1^ m^−1^) was obtained in both species by dividing the total energy expended during the swim by the total distance the animal swam during the trial. Validation of stroke detection as well as distance traveled was through visual and video recorded observations. Videos were taken using an overhead surveillance camera (Avigilon, Dallas, TX, USA) and analyzed using the Tracker Video Analysis and Modeling Tool (Tracker 5.0.7, Open Source Physics). Observers at underwater viewing windows also monitored stroke frequency and swim tracks of submerged swimming animals. Swim speed (m s^−1^) was calculated by dividing the total distance swum by the total submergence duration.

For dynamic acceleration metrics, static (i.e. gravitational) acceleration was calculated using a 2 s running mean of the raw acceleration (Wilson et al., 2006). This static value was subtracted from the total raw acceleration, and the absolute value was calculated as the dynamic acceleration. Partial dynamic body acceleration (PDBA, ***g***) of the individual axes was also calculated as the mean dynamic acceleration of the axis during the submergence period as in Wilson et al. (2006). To incorporate the acceleration from multiple axes (*x*, *y* and *z*) simultaneously, PDBA of paired combined axes was calculated from the mean accelerations during the submergence period as based on the sum of the dynamic acceleration from two individual axes. In addition, overall dynamic body acceleration (ODBA, ***g***), representing the mean acceleration during the submergence period, was determined from the sum of the dynamic acceleration from all three individual axes (Halsey et al., 2009; Wilson et al., 2006).

#### Analyses

A mixed-effect repeated measures model was used to compare metabolic rates during fed and fasted swims. Linear mixed models were subsequently used to examine the relationship between *V̇*_O_2_ss_ and submergence duration, swim speed, stroke frequency and acceleration in beluga whales. Owing to the same individuals being sampled multiple times, individual was treated as the random effects subject in the repeated measures approaches. Single-axis, two-axis and three-axis accelerometer metrics were calculated and analyzed to determine the most robust relationship between *V̇*_O_2__ and acceleration as indicated by AICc and BIC scoring. These analyses were conducted in R (https://www.r-project.org/) and JMP Pro (Version 14.3.0, SAS Institute Inc., Cary, NC, USA). All compiled results are presented as means±s.e.m unless otherwise noted.

Comparative analyses of metabolic rates and energetic costs with other species of marine mammals were based on a Kleiber (1975) approach utilizing a specific list of testing criteria to account for variance. Criteria included the original Kleiber conditions for thermoneutrality, age and fed state, as well stationing behavior on the water surface for rest, common open-flow respirometry methods and species-specific testing durations to accommodate potential apneic periods during rest and recovery. Thus, rather than a comprehensive comparison of all available studies as previously conducted (i.e. Davis, 2019; Rimbach et al., 2021), we focused on a subset of studies with identical protocols to identify potential species-specific differences.

## RESULTS

### Resting metabolic rates

**Fig. 2.**
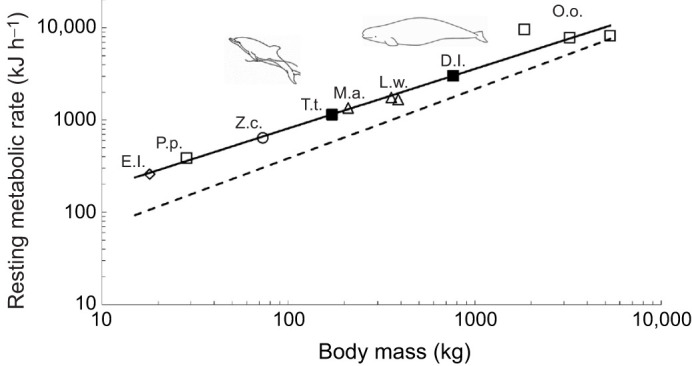
**Resting metabolic rate in relation to body mass for marine mammals.** The solid line is the allometric regression for marine mammals stationing on the water surface adapted from Williams et al. (2001) where, RMR=41.509*M*^0.65^ for 8 species. The dashed line is the predicted regression for domestic terrestrial mammals as described by Kleiber (1975). The closed squares represent mean RMR for beluga whales (D.l., present study) and bottlenose dolphins (T.t., present study); both are within 2% of the predicted RMR for marine mammals. Open symbols represent mean RMR for sea otters (E.l. diamond; Williams, 1989), harbor porpoises (P.p. square, Kanwisher and Sundnes, 1965), California sea lions (Z.c. circle, Liao, 1990), northern elephant seals (M.a. triangle, Costa et al., 1986), Weddell seals (L.w. triangle, Castellini et al., 1992; Williams et al., 2004b) and killer whales (O.o. square, Kriete, 1995; Williams et al., 2017b; Worthy et al., 2014). Marine mammal species presented in this comparison typically reside in cold temperate or polar environments, and were measured with open-flow respirometry at routine water temperatures for each species.

Average RMR for all three beluga whales was 3012±126.0 kJ h^−1^ (*n*=39 trials; Table 2, Fig. 2). Mass-specific RMR across the three whales averaged 65.8±2.73 J kg^−1^ min^−1^ (*n*=39 trials; Table 2). As would be expected based on smaller body mass (Kleiber, 1975; McNab, 2008), the average whole-body RMR for the two bottlenose dolphins measured in this study was lower than for the beluga whales at 1140±15.9 kJ h^−1^ (*n*=37 trials; Fig. 2). Mass-specific RMR for both dolphins was 111±1.7 J kg^−1^ min^−1^ (*n*=37 trials; Table 2), and higher than that of the beluga whales as would be anticipated from allometric theory.

**Table 2.**
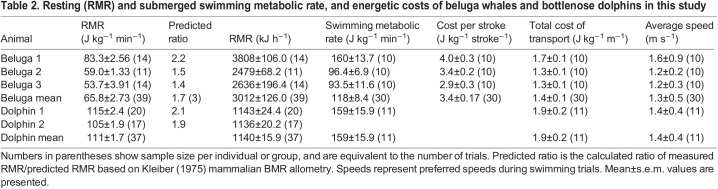
Resting (RMR) and submerged swimming metabolic rate, and energetic costs of beluga whales and bottlenose dolphins in this study

### Swimming metabolic costs

**Fig. 3.**
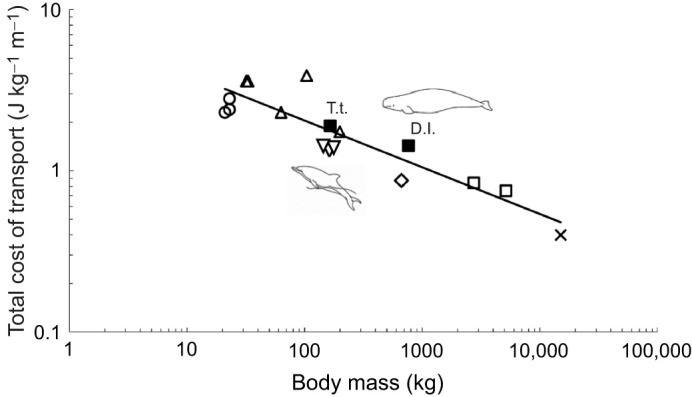
**Total cost of transport in relation to body mass for beluga whales in relation to other swimming marine mammals.** The closed squares represent mean values for beluga whales (D.l.) and bottlenose dolphins (T.t.) in the current study. Comparative data are presented in open symbols for phocid seals (upright triangles; Davis et al., 1985; Fedak, 1986; Williams et al., 1991; John et al., 2021), California sea lions (circles; Feldkamp, 1987; Williams, et al., 1991; Williams, 1999), West Indian manatees (diamond; John et al., 2021), other bottlenose dolphins (downward facing triangle; Williams et al., 1993; van der Hoop et al., 2014; Allen et al., 2022), killer whales (open squares; Kriete, 1995) and grey whales (estimated from respiratory rates; X; Sumich, 1983). The solid line is the allometric regression for COT_TOT_ during continuous submerged swimming in marine mammals as adapted from Williams (1999) for *n*=9 species, including original mean data for three beluga whales and two bottlenose dolphins in the present study.

We found no significant difference in the cost of submerged swimming during fasted or fed states for the beluga whales in this study based on small meal sizes (*n*=30 swims, d.f.=1, 26.3, *F*=0.067, *P*=0.7976). This was not surprising owing to the timing of feeding relative to metabolic trials, and the comparatively small heat increment of feeding response (<22% increase in oxygen consumption) of moderately sized odontocetes (Yeates and Houser, 2008). Therefore, the metabolic data are combined here. Preferred swimming speed for the beluga whales averaged 1.3 m s^−1^ (range: 0.9–1.9 m s^−1^). At this speed, the mean energetic cost of submerged swimming was 118±8.4 J kg^−1^ min^−1^ across all three whales (*n*=30 swims), approximately two times resting values. Average cost per stroke was 3.4±0.2 J kg^−1^ stroke^−1^ (*n*=30 swims). COT_TOT_ based on this stroking cost and stroke number averaged 1.4±0.1 J kg^−1^ m^−1^ (*n*=30 swims), approximately 27% higher than predicted for similarly sized marine mammals (Williams, 1999; Table 2, Fig. 3). The mean preferred swimming speed for the bottlenose dolphin measured in this study was 1.4 m s^−1^ (range: 1.2–2.1 m s^−1^). At this speed, the energetic cost of submerged swimming for dolphin 1 was 159±15.9 J kg^−1^ min^−1^ (*n*=11 swims). COT_TOT_ for the dolphin was 1.9±0.2 J kg^−1^ m^−1^ (*n*=11 swims), within approximately 6% of the predicted value (Table 2, Fig. 3).

### Predicting energetic output

**Fig. 4.**
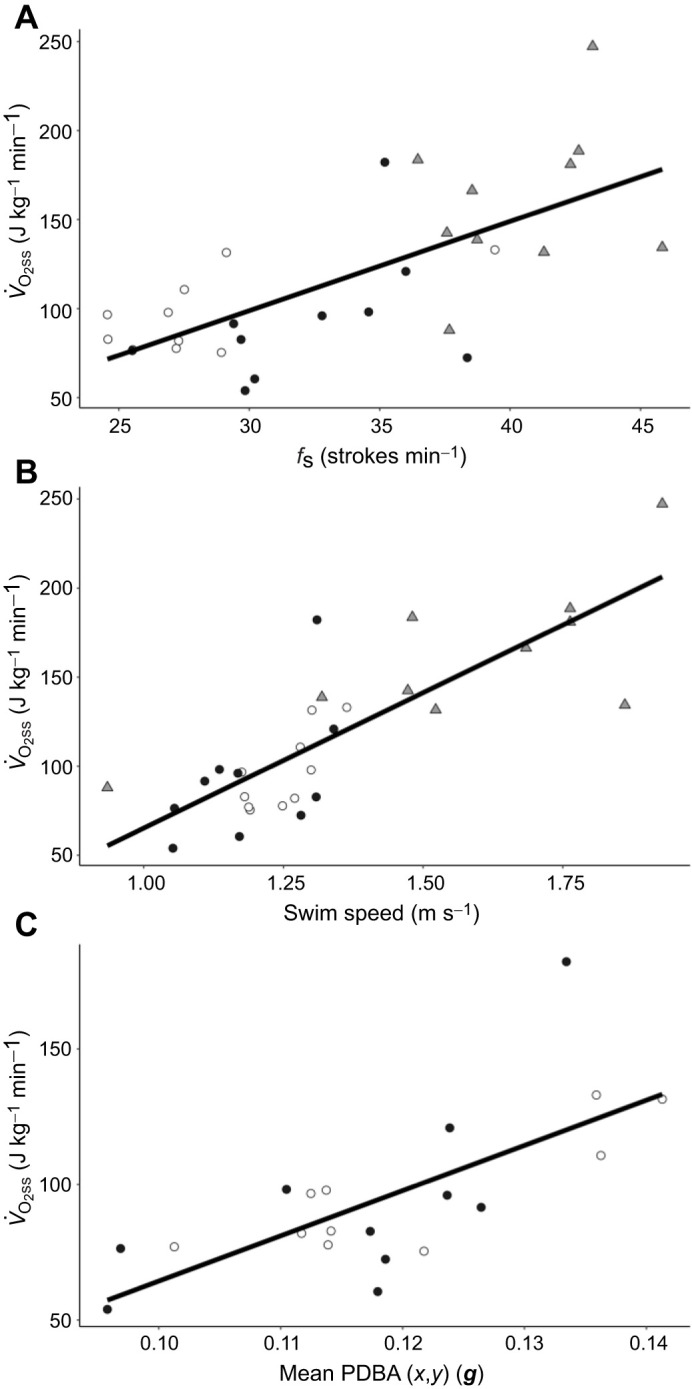
**The rate of oxygen consumption during submerged swimming (*V̇*_O_2_ss_) in beluga whales.** *V̇*_O_2_ss_ data are presented in relation to (A) stroke frequency (*f*_S_), (B) swim speed and (C) mean partial dynamic body acceleration [PDBA(*x*,*y*)]. Each point represents the average value and rate of oxygen consumption for a single submerged swim by an individual animal in this study. Beluga 1 (grey triangles), beluga 2 (white circles) and beluga 3 (black circles) are compared. The solid lines are the least squares linear regressions for all animals as described by Eqns 1–3 in the text. Data are presented for three whales in A and B, and only two whales in C as detailed in the Discussion.

To determine the effect of locomotor characteristics on energetic costs in beluga whales, we examined the correlation between *V̇*_O_2_ss_ (oxygen consumption during submerged swimming) and submergence duration, stroke frequency, swim speed and acceleration. There was no significant relationship between submergence duration (mean 177±6.9 s; range: 119–233 s) and mass-specific *V̇*_O_2_ss_ (*n*=26 swims, *r*^2^=0.03, *P*=0.36); this is as expected when comparing total submergence duration and the rate of energy expended per minute for these relatively short swims. Conversely, as found for bottlenose dolphins (Williams et al., 2017b), there were highly significant relationships between *V̇*_O_2_ss_ (J kg^−1^ min^−1^) and *f*_S_ (strokes min^−1^) (*n*=30 swims, d.f.=1, 28, *F*=25.53, *P*<0.0001, AICc=304.28, BIC=308.29; Fig. 4A) for beluga whales, where:
(1)

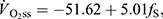
and between *V̇*_O_2_ss_ (J kg^−1^ min^−1^) and swim speed (*U*_swim_, m s^−1^) (*n=*30 swims, d.f.=1, 28, *F*=54.93, *P*<0.0001, AICc=288.48, BIC=291.76; Fig. 4B), where:
(2)

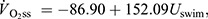
even within the relatively narrow range of exercise efforts representing the preferred performance levels of the individual whales. This contributed to higher or lower preferred stroke frequencies for individuals (Fig. 4A) that spanned the range of dynamic acceleration outputs (Fig. 4C).

Additionally, we found significant linear relationships between *V̇*_O_2_ss_ and multiple dynamic acceleration metrics (John, 2020), with the most robust relationship occurring for *V̇*_O_2_ss_ in J kg^−1^ min^−1^ and PDBA(*x*,*y*) in ***g*** (*n=*20 swims, d.f.=1, 18, *F*=18.19, *P*=0.0005, AICc=184.77, BIC=186.26; Fig. 4C), where:
(3)

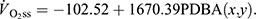


## DISCUSSION

The challenge of predicting energetic requirements and resource needs for wild cetaceans begins with our ability to accurately determine the metabolic foundations of each species. With a remarkable 2200-fold range in cetacean body mass from 55 kg vaquita (*Phocoena sinus*) to 122,000 kg blue whales (*Balaenoptera musculus*), generalizing metabolic patterns and other physiological processes across all dolphins and whales is nearly impossible (Noren and Williams, 2000). This is especially problematic for moderately sized cetaceans, such as beluga whales, that may fall into high energetic cost patterns typical of smaller cetaceans during months associated with reproduction, long transits and intensive foraging (Gallagher et al., 2021), or into low energetic demand–cost-efficient modes characteristic of the largest whales during less active periods (Lockyer, 2007). Here, we found that the energetics of non-reproductive, adult beluga whales generally followed energetically intensive resting metabolic and locomotor cost patterns similar to those predicted for like-sized marine mammals living in cold environments, including larger dolphins and female killer whales (Williams et al., 2001, 2017b).

### Variability in resting metabolic rate of beluga whales compared with dolphins

The RMRs of adult beluga whales and dolphins in the present study were higher than those predicted for terrestrial mammals of similar body mass (Kleiber, 1975; McNab, 2008) (Fig. 2), following the trend of elevated metabolic demands reported for small to moderate-sized, cold-water adapted marine mammals (Davis, 2019). For bottlenose dolphins, this elevated RMR was within 18% of levels reported by Yeates and Houser (2008) when comparing similarly-sized dolphins, and agrees with the conclusions of Yazdi et al. (1999) and van der Hoop et al. (2014) also using open-flow respirometry with fasted animals. All four marine mammal studies, as well as those summarized in Yazdi et al. (1999), indicate elevated RMRs in dolphins relative to predicted basal metabolic rate (BMR) from Kleiber (1975), ranging from 1.5 to 2.5 times predicted in the previous studies and 1.9 to 2.1 predicted in the present study (Table 2).

These results differ from Allen et al. (2022) using breath-by-breath spirometry with a pneumotachometer placed over the animal's blowhole. Further research is needed to determine the significance of these protocol differences. That said, evidence at the sub-cellular level suggests that heightened thermogenic mitochondrial leak and regulated heat production in the skeletal muscles of small and moderate-sized marine mammal species may explain in part the basis for an elevated RMR (Wright et al., 2021), which may also apply to some cetaceans (Williams, 2022). Interestingly, the allometric relationship for RMR in marine mammals appears to converge with the Kleiber (1975) relationship for exceptionally large species, suggesting that differences in RMR between mammalian groups may diminish as whale size increases and related thermoregulatory challenges decrease (Favilla et al., 2023; Fig. 2). Similarly, a meta-analysis by He et al. (2023) reported that differences in allometry for basal metabolic rate of large and small marine mammals compared with terrestrial mammals may be explained in part by parallel differences in the allometry of cardiovascular elements within the oxygen pathway.

The comparatively elevated RMRs of the three beluga whales measured here (mean=1.71±0.25 times Kleiber predications for mammals; Table 2) are similar to values reported by Kasting et al. (1989). Lower values were reported by Rosen and Trites (2013) for a 17-year-old male beluga whale despite using identical methodologies. The latter whale at 1341 kg and heavily blubbered was markedly larger than the average beluga whale, prompting the authors to suggest that its comparatively low metabolic rate may be related to a proportionately lower lean-to-fat mass ratio (i.e. increased amount of metabolically inactive blubber relative to total mass). This has also been observed for northern elephant seal pups, where *V̇*_O_2__ can correlate more strongly to lean body mass than total body mass (Rea and Costa, 1992).

This variability in mass-specific metabolism associated with changes in fat mass suggests that care is needed when measuring and reporting metabolic rates in beluga whales. Given the marked seasonal changes in body condition observed for this cetacean species (Breton-Honeyman et al., 2016), metabolic rates should be viewed relative to extreme changes in blubber deposition and lean body mass. This is especially important when trying to model thermal demands and food requirements of wild whales based simply on predictive relationships between body mass and metabolic rate. Thinner whales, whether due to age, overall body size or seasonal depletion of body lipid stores, have a proportionately greater amount of metabolically active tissue compared with larger, more robust whales, and thus greater potential for relative heat loss (Favilla et al., 2023). Doidge (1990) has demonstrated how this can affect theoretical resting metabolic heat production and heat loss calculated from total body mass or from body mass excluding blubber. Note, however, that a recent study examining brown adipose tissue in cetacean blubber indicates that this tissue may not be as metabolically inert as originally assumed (Hashimoto et al., 2015), tempering metabolic corrections necessary to account for thin or robust whales except in extreme cases. Thus, for the purposes of the present study, the RMR of the beluga whales measured here should be considered a reasonable baseline with no further modifications required to account for excess lipid deposition.

### Active metabolic rates of beluga whales: the cost of surface and submerged swimming

Cook Inlet beluga whales typically inhabit shallow coastal waters, and, as such, spend over 78% of their time swimming at or near the surface (<1 m depth) compared with 21.6% of their time diving (Goetz et al., 2012a,b). This makes the cost of surface activity a potentially significant part of maintaining energy balance in this species. We found that locomotor costs of adult beluga whales during submerged swimming in shallow water were consistent with predicted values for moderately sized (200–1000 kg) marine mammals, including a COT_TOT_ for belugas that was within 27% of the expected value for other swimming marine mammals (Fig. 3).

These measured locomotor costs for submerged swimming combined with RMR measured for sedentary beluga whales on the water surface allow us to specifically model the average cost of surface activity in this species. To begin, a general approximation of FMR for marine mammals including odontocetes (Noren et al., 2024) is three times RMR (Noren, 2011; Williams et al., 2004a, 2020; Williams, 2022). A more precise calculation for beluga whales based on measured and estimated values for marine mammals from 27 kg sea otters to 3886 kg killer whales can be determined from:
(4)

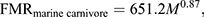
where FMR is in kJ day^−1^ and body mass (*M*) is in kg (*n*=14 species, *r*^2^=0.84, *P*<0.001) (from Williams et al., 2020). As an example, based on the average mass of adult beluga whales in the present study (758 kg; Table 1), this equation yields a predicted FMR of 208,462 kJ day^−1^ (49,822 kcal day^−1^). This sets the theoretical average total daily metabolic expenditure of the beluga whale. With a measured submerged swimming metabolic rate of 118.0 J kg^−1^ min^−1^ (Table 2) and an average time spent submerged each day of 21.6% (311 min) reported for Cook Inlet beluga whales (Goetz et al., 2012b), this results in a total metabolic cost of 27,817 kJ day^−1^ expended for submerged swimming. Lastly, the difference between the calculated FMR and daily submerged swimming costs determined here (208,462–27,817 kJ day^−1^) results in a total daily cost for surface activity of 180,645 kJ day^−1^ or 165.5 J kg^−1^ min^−1^. This value is approximately 40% greater than submerged swimming costs measured here. Several factors may have contributed to this difference, including the increased hydrodynamic drag of marine mammals while on the surface (Williams, 1989; Fish, 1994), a reduction in gliding periods when traveling horizontally (Davis, 2019), and a release from the dive response influencing submerged swimming metabolism (John et al., 2021).

In view of these results, knowing the proportion of each day that beluga whales spend active at or near the surface rather than diving will be important when calculating FMR. This is especially relevant when the animals alter behavior in response to anthropogenic disturbances. For example, beluga whales avoiding a threat by transiting near the water surface may incur a higher energetic cost than escaping by diving. Much of the difference in the resulting costs will depend on the ability of the animal to incorporate periods of cost-efficient gliding during a dive, as reported for a related odontocete, the narwhal (*Monodon monoceros*) (Williams et al., 2017a). Narwhals exposed to anthropogenic noise showed an increase in the cost of both transit swimming and diving that was mediated by marked increases in stroke frequency during escape, especially when diving (Williams et al., 2022). Obviously, for the Cook Inlet beluga whale population, increased knowledge of the preferred locomotor responses, including position in the water column and stroking patterns during a dive, will increase the accuracy of such energetic assessments.

### Predicting energetic costs

The animal-borne accelerometers deployed on belugas in the present study demonstrated the level of interplay between energetic costs and stroking effort. Strong correlations were found between *V̇*_O_2_ss_ and all locomotor metrics (*f*_S_, swim speed and body acceleration) during submergence. Importantly, although *f*_S_, swim speed and combined *x*- and *y*-axis (dorso-ventral) dynamic acceleration each resulted in significant relationships, stroke rate and swim speed included 50% more samples as one whale was not measured using the accelerometer tag. When swim speed and *f*_S_ are considered only from the animals with concurrent accelerometer measurements, the predictive strength of both variables decreases relative to accelerometry. Swim speed remains a significant predictor of *V̇*_O_2_ss_ (*n=*20 swims, d.f.*=*1,18, *F*=9.48, *P*=0.0065, AICc=190.28, BIC=194.94); however, *f*_S_ no longer yields a significant relationship with just the two animals (*n=*20 swims, d.f.*=*1,18, *F*=3.76, *P*=0.0682, AICc=191.76, BIC=196.43). As a result, this study indicates a stronger correlation between acceleration and energetic cost for individuals, whereas both stroke rate and speed are additional robust predictors of energetic cost during a dive for animal groups (see John, 2020 for further details of the comparative analyses). Admittedly, a broader sampling of animals, including young, pregnant, lactating and seasonally fattened individuals, will likely reveal variance around these predictive metrics, and awaits further study.

These locomotor metrics, determined on trained animals, provide a unique opportunity to assess metabolic rates of free-ranging beluga whales with appropriate instrument calibration. As discussed above, this involves accounting for the difference in energetic costs between surface and submerged swimming. Because surface swimming incurs a 40% increase in energetic cost over submerged swimming (based on the proportion of daily energetic costs calculated above), Eqns 1–3 used for determining *V̇*_O_2_ss_, the cost of submerged swimming, can be rewritten incorporating a correction factor as:
(5)

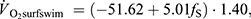

(6)

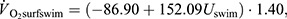

(7)

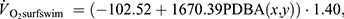
to predict the cost of surface swimming (*V̇*_O_2_surfswim_ in J kg^−1^ min^−1^) from stroke frequency, swimming speed and acceleration with the same units as Eqns 1–3, respectively. The correction factor accounts for potential differences in maintenance costs of the animals on the water surface or submerged as a result of changes in the dive response. Furthermore, it affords higher resolution when partitioning total costs expended by this species during different locomotive modes and activity states that comprise the daily activity budgets of this cetacean. Because the physiological response to diving in cetaceans is modified by both dive depth and exercise intensity (Davis and Williams, 2012; Williams et al., 2015), additional modifications to costs may be anticipated with exceptionally deep dives or high levels of exercise performance.

### Assessing aerobic dive limits in beluga whales

**Fig. 5.**
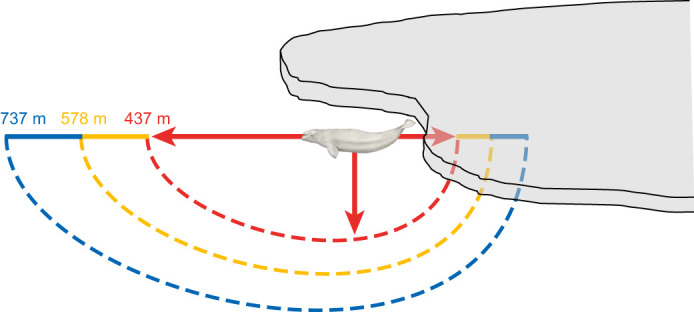
**Distance limits for aerobic submerged activity by beluga whales.** Colors correspond to slow (1.3–1.4 m s^−1^, blue line), moderate (1.9 m s^−1^, yellow line) and fast (7.6 m s^−1^, red line) swimming speeds relative to measured routine speeds. Grey area on the right represents sea ice cover, with the trajectories (indicated by the arrows) and relative excursion distances (indicated by the colored dashed lines) modified by the on-board oxygen stores of adult belugas for each speed range. Note that deeper transects will reduce the linear horizontal distance travelled, and that the data are for one-way excursions requiring a breathing hole at the end. Consequently, sub-ice distance limits would be halved if the animal has to return to the original breathing hole.

In addition to the energetic costs associated with resting, swimming and diving, the aerobic dive limit (ADL) remains an important metric for understanding the capacity of this relatively deep-diving whale to remain submerged (Kooyman et al., 1980; Davis et al., 2013). For Arctic and subarctic species such as the beluga whale that must contend with changing sea ice cover, the ADL helps to define how much of the sub-ice environment is available for travel, foraging and escape (Williams et al., 2011b, 2017a). Using our measurements for the average cost of submerged swimming and assuming a mass-specific oxygen store of 51 ml O_2_ kg^−1^ (Shaffer et al., 1997), the calculated aerobic dive limit (cADL) for beluga whales is 8.8 min. This is comparable to the 9 min cADL reported by Shaffer et al. (1997) based on measurements of post-dive plasma lactate concentration in free-ranging, trained beluga whales. At an average swimming speed of 1.3–1.4 m s^−1^ (Table 2; Shaffer et al., 1997), this translates into a maximum linear sub-ice swimming distance of 737 m to avoid transitioning to anaerobic metabolism with a concomitant accumulation of lactate byproducts (Fig. 5).

Because oxygen consumption increases with swim speed in beluga whales (Fig. 4), an elevation in speed in response to unanticipated disturbance (see Williams et al., 2017a,b, 2022) will result in faster depletion of onboard oxygen stores. For example, the highest speed measured in this study was 1.9 m s^−1^. Using Eqn 2, the calculated *V̇*_O_2_ss_ for this effort is 202.1 J kg^−1^ min^−1^ or 10.1 ml O_2_ kg^−1^ min^−1^. In this case, cADL would decrease from 8.8 to 5.1 min, and the linear swim distance covered in a single dive would decrease from 737 to 578 m (Fig. 5). Performing the same calculations for the maximum speed measured in beluga whales (7.6 m s^−1^; Richard et al., 1998) yields a cADL of just 1.0 min and a maximum linear swim distance of only 437 m. Note that this is an extrapolation beyond the routine speeds measured in the present study, and does not account for the non-linear increase in oxygen consumption typically reported for some fast-swimming marine mammal species (Williams et al., 2017b). As a result, our calculations should be considered conservative estimates of the rate at which beluga whales expend onboard oxygen stores during extreme flight behaviors. Additionally, recognize that this is a one-way excursion, requiring an accessible open area in the ice to accommodate breathing and post-dive replenishment of oxygen stores. Absence of an open area would require a reverse in direction at the half-way outpoint to reach the original breathing hole while remaining aerobic.

### Energetic profiles of Cook Inlet beluga whales

Beluga whales in Cook Inlet, Alaska, are exposed to multiple, concurrent stressors as a result of their proximity to populated areas (NMFS, 2016). These include threats from catastrophic events such as oil spills, chronic noise pollution associated with increased development and shipping traffic, as well as reductions in access to prey. Similar threats are also a concern for other populations of belugas living in Arctic and subarctic regions, where declining sea ice cover is opening larger areas for increased human activities (Laidre et al., 2008; NAMMCO, 2022). To anticipate and mitigate the effects of such anthropogenic stressors, the energetic measurements detailed in the present study allow scientists and wildlife managers to begin to evaluate the capacity of this species to respond both behaviorally and physiologically (Fig. 5).

To summarize, we found that beluga whales often exhibited an energetically intensive lifestyle. RMRs were elevated compared with those of terrestrial mammals, and within 1% of the predicted RMR for similarly sized marine mammals residing in cold temperate or polar environments (Fig. 2). The propensity of this species to spend the majority of its time near the water's surface (especially in the summer months, when foraging on salmon at river mouths; NMFS, 2016) also contributes to elevated energetic costs. Based on our calculations, the cost of surface swimming was 40% greater than submerged. Consequently, we provide a correction factor (Eqns 5–7) for energetic models to account for this difference in costs when determining daily field metabolic rates involving positional changes in the water column during escape/avoidance responses. Lastly, using the average cost of submerged swimming measured in this study, we calculated an ADL of 8.8 min for belugas that drives movement decisions. At preferred swimming speeds, this cADL translates into a maximum linear submerged swimming distance of 737 m to remain aerobic, with faster depletion of oxygen stores and marked decreases in cADL and travel distances with higher speeds that can occur when reacting to disturbance. A theoretical extrapolation to maximum speed, indicates that cADL may be reduced to 1.0 min, resulting in a linear swim distance <60% of normal. It is likely that this is a conservative estimate of the actual impact on fleeing belugas owing to anaerobic costs incurred at high levels of exercise (Shaffer et al., 1997).

This energetics approach has proven effective for understanding the impact of human disturbance on other marine mammals (Costa et al., 2016; McHuron et al., 2017, 2018; New et al., 2013; Pirotta et al., 2018b; Williams et al., 2017a, 2022). For example, using energetics data from the present study combined with recordings from accelerometer–depth tags, Williams et al. (2022) estimated a 2- to 3-fold increase in the cost of escape dives relative to undisturbed dives by another Arctic odontocete, the narwhal, following exposure to seismic activities. Further research on wild belugas instrumented with accelerometer–depth recorders will be needed to determine whether they are similarly affected.

In addition to developing locomotor energy budgets, the basic measurements presented here also allow biologists to estimate the requirement for salmon, a key prey item of Cook Inlet beluga whales (Goetz et al., 2012a,b; Quakenbush et al., 2015), by balancing energy expenditures against energy acquisition. Using our FMR values (based on body mass, Eqn 4) and assuming a digestive efficiency (DE) of 0.85% similar to that of killer whales (Williams et al., 2011a), an energy balance equation is:
(8)

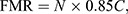
where FMR is in kcal day^−1^, *N* is the number of fish per day and *C* is the caloric value (kcal) per fish. This is converted to:
(9)

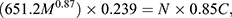
where *M* is beluga mass in kg and predicted FMR is multiplied by a constant to convert to kcal day^−1^. To solve for *N* (the number of fish needed each day to satisfy the energetic demands of a beluga whale) requires information about the caloric value of prey fish. For salmon, this is available from Noren (2011) and O'Neill et al. (2014). In our example, an average adult beluga whale weighing 758 kg (Table 1) would need to consume four to five chinook ‘king’ salmon (*Oncorhynchus tshawytscha*) per day to satisfy an FMR of 49,822 kcal day^−1^ (calculated from 13,409 kcal fish^−1^×0.85 to account for DE, which results in 11,398 kcal digested for each fish; O'Neill et al., 2014). By comparison, the individual whale would require 12 coho salmon (*Oncorhynchus kisutch*, 4982 kcal fish^−1^) per day, or 13–14 chum salmon (*Oncorhynchus keta*, 4265 kcal fish^−1^) or sockeye salmon (*Oncorhynchus nerka*, 4264 kcal fish^−1^) per day. With lower reported energy contents per fish, it takes 28 pink salmon (*Oncorhynchus gorbuscha*, 2101 kcal fish^−1^) per day to balance daily energy costs of the beluga whale (an online beluga fish calculator for these and other fish can be found at https://enazario11.github.io/beluga_fish_calculator/Beluga_fish_calculator.html).

Obviously, species-specific and seasonal variation in the body mass and lipid content of each type of salmon affects the caloric value of individual fish (O'Neill et al., 2014), and thus the demand by belugas. Prey choice by beluga whales will also differentially impact these salmonid populations, and ultimately the availability of individual salmon species needed to support the recovery of a robust Cook Inlet beluga whale population.

By routinely evaluating energetic balance in these whales, the potential for specific prey resources to seasonally represent a limiting factor in species recovery is possible. As demonstrated here, such energetic balance sheets provide a mechanism for refining predictions regarding the response, recovery and resiliency of individual beluga whales that can be scaled to larger populations. The availability of these predictive tools is particularly timely as declines in sea ice cover accelerate, allowing ever increasing movements of humans into the Arctic and subarctic that will directly affect this species, its prey and the environment.
